# Population Characteristics of the Spectrum and Frequencies of *CFTR* Gene Mutations in Patients with Cystic Fibrosis from the Republic of Bashkortostan (Russia)

**DOI:** 10.3390/genes15101335

**Published:** 2024-10-17

**Authors:** Guzel Ayupova, Sergey Litvinov, Vita Akhmetova, Ildar Minniakhmetov, Natalia Mokrysheva, Rita Khusainova

**Affiliations:** 1 Department of Medical Genetics and Fundamental Medicine, Bashkir State Medical University, 450008 Ufa, Russia; guzel8319@gmail.com; 2 Institute of Biochemistry and Genetics, 450000 Ufa, Russia; seregtg@gmail.com (S.L.); vita-akh@mail.ru (V.A.); 3 Endocrinology Research Centre, 117292 Moscow, Russia; minniakhmetov.ildar@endocrincentr.ru (I.M.); mokrisheva.natalia@endocrincentr.ru (N.M.)

**Keywords:** cystic fibrosis, CFTR, population genetics, newborn screening, CFTR mutations, DNA diagnostics, algorithms of genetic counseling

## Abstract

Background/Objectives: Cystic fibrosis (CF) is one of the most common autosomal-recessive disorders worldwide. The incidence of CF depends on the prevalence of cystic fibrosis transmembrane conductance regulator gene (*CFTR*) mutations in the population, which is determined by genetic diversity and ethnicity. Methods: The search for the causes of mutations in the transmembrane conductance regulator gene (CFTR) was carried out using targeted next-generation sequencing (NGS) on the Illumina platform in patients with cystic fibrosis from the Republic of Bashkortostan (Russia), taking into account the ethnic structure of the sample. Results: A total of 35 distinct causal variants were found in 139 cases from 129 families. Five (F508del, E92K, 3849+10kbC>T, CFTRdele2.3, L138ins) explain 78.7% of identified CF causal alleles. Variants N13103K and 394delTT were found in four families each. Variants 2143delT, S1196X, W1282X, Y84X, G194R, and 1525-1G>A, as well as the two previously described complex alleles—c. [S466X; R1070Q] and str.[G509D;E217G]—were found in two or three families each. Twenty additional variants occurred only once. Variant c.3883_3888dup has not been described previously. Thus, regional and ethnic features were identified in the spectrum of frequencies of pathogenic variants of the CFTR gene in the three major sub-groups of patients—Russians, Tatars, and Bashkirs. Conclusions: Taking into account these results, highlighting the genetic specificity of the region, a more efficient search for CFTR mutations in patients can be performed. In particular it is possible to choose certain test kits for quick and effective genetic screening before use of NGS sequencing.

## 1. Introduction

Cystic fibrosis (CF) is one of the most common autosomal-recessive disorders worldwide. The incidence of CF depends on the prevalence of cystic fibrosis transmembrane conductance regulator gene (*CFTR*) mutations in the population, which is determined by genetic diversity and ethnicity. Although one mutation remains the most common cause of CF (F508del), there have been more than 2000 reported variations in *CFTR* and there are population characteristics in the prevalence of pathogenic changes in the gene in patients from different geographic regions of the world. It is well known that F508del is the most common mutation worldwide [1]. This is also true for the Russian population, but as for the other mutations, their spread varies in different ethnic groups from Russia [2]. This imposes certain difficulties for the medical services responsible for the quick and reliable detection of pathogenic alleles in patients. It has been already taken into account that population genetics and penetrance data are important for the variant impact evaluation [3]. Another problem is also well known, particularly for association studies, and is related to the European genetic ancestry bias when the number of candidate variants is less in geographic ancestry groups other than European [4].

Although the treatment of cystic fibrosis (CF) is one of the actual issues because of its prevalence, establishing a diagnosis is still a challenge, and molecular diagnosis related to the search for mutations can still be a problem because of their specific distribution across different populations [3]. The population of the Republic of Bashkortostan, according to Rosstat, is 4,064,361 (January 2024). This region bridges the European part of Russia with Siberia and Central Asia, and represents an important crossroads for active labor migration. The population density is 28.08 people/km^2^ (2021). Representatives of 160 nationalities and 13 ethnic groups that make up the population live in the territory of Bashkortostan; the most numerous ethnic groups remain Russians (36 percent) belonging to the Slavic group of the Indo-European language family, Bashkirs (29.5 percent), and Tatars (25.4 percent), the Turkic group of the Altai language family, respectively. This is followed by the Chuvash (2.7 percent), Mari (2.6), Ukrainians (1), Udmurts (0.5), Mordovians (0.5), and Belorussians (0.3).

While the situation for autosomal-recessive disorders is better, it is not always clear how to plan the search for mutations in the representatives of less-studied populations. It is especially quite important when there is a patient with an established diagnosis in a family and the pathogenic alleles are not known to conduct prenatal diagnostics in the future. To solve such an issue, a mutation spectrum in separate ethnic groups from different regions must be defined to arrange an algorithm for *CFTR* analysis in each case. NGS has been shown to be an effective instrument for molecular testing in severe childhood recessive diseases [5], and in CF patients in particular [6,7], including for pre-implantation purposes [8]. It also showed good results when analyzing CF in rather admixed populations from Brazil [9] and infertile patients from the Sicilian population, which showed heterogeneity for the *CFTR* mutations [10]. Applying this approach to the populations from the Volga region and the Urals, which showed a motley genetic landscape [11,12], showed that it is a suitable method for both diagnosis confirmation and revealing the true mutation spectrum for the subsequent quick CF allele search with Sanger sequencing.

The aim of this study was to search for a full range of mutations in the transmembrane conduction regulator (*CFTR*) gene in patients with cystic fibrosis from the Republic of Bashkortostan (Russia), taking into account the population structure of the region, in order to optimize DNA diagnostics and genetic counseling algorithms.

## 2. Materials and Methods

Venous blood samples of 139 patients from 129 families from the Republic of Bashkortostan, as well as of 250 members of their families (parents and siblings), were collected. In five families, two children were studied. The largest number of families were of Russian origin (43.4%), followed by Tatars (29.5%) (Table 1). The next most common were admixed individuals, mainly from marriages between Russians and Tatars (9 families), and Tatars and Bashkirs (5 families), as well as several cases of marriages between Russians, Uzbeks, Poles, Belorussians, Chuvashes, Tatars, and Bashkirs.

All samples were collected from 1998 to 2023. The cystic fibrosis diagnosis was established for 89 patients, and 50 patients were either suspected for CF based on clinical data or were included in the study based on the results of neonatal screening.

The ethnic composition of the patients was established based on questionnaires and information on ancestry up to the third generation. Based on the information obtained and DNA analysis by Sanger sequencing, all identified mutations were distributed across chromosomes in accordance with the ethnic origin of the parents, even in the case of a mixed marriage, and an analysis of the frequencies and spectrum of mutations was carried out taking into account the ethnic origin of the chromosome with the identified mutation. Samples from the siblings of the patients were also studied by Sanger sequencing.

For clinical definitions, Clinical Recommendations “Cystic fibrosis” approved by the Russian Ministry of Health were used: classical cystic fibrosis with pancreatic insufficiency (mixed or pulmonary-intestinal form of the disease, PI. E84.8), classical cystic fibrosis with undisturbed pancreatic function (predominantly pulmonary form of the disease, PS. E84.0), uncertain diagnosis with positive neonatal screening for cystic fibrosis (E84.9), diseases associated with the gene CFTR(MVTR): isolated obstructive azoospermia, chronic pancreatitis, and disseminated bronchiectasis.

The study was approved by the Bioethics Committee of the Bashkir State Medical University, protocol No. 11 dated 16 December 2020. All patients and healthy donors provided signed informed voluntary consent to participate in the study in accordance with the Helsinki Declaration of the World Medical Association “Ethical Principles for Medical Research Involving Human Subjects”.

DNA was extracted using a QIAamp DNA Blood Mini Kit (Qiagen, Hilden, Germany) and diluted in water. The final amount of DNA was brought to 10 ng per reaction, and the concentration was verified with a Qubit^®^ 3.0 Fluorometer (Thermo Fisher Scientific, Inc., Waltham, MA, USA). Next-generation sequencing (NGS) technology was used to search for pathogenic variants of the CFTR gene on the Illumina platform (MiSeq, San Diego, CA, USA). For the NGS library preparation, we used three reagent kits: “VariFind™ Neoscreen assay” and “VariFind™ CFTR solution” (Parseq Lab, Saint Petersburg, Russia) for the target enrichment, and “Prep&Seq™ U-target IL library kit” (Parseq Lab) for the end preparation, adapter ligation, library amplification, and purification using the official protocol. The libraries were then sequenced on Illumina MiSeq using a MiSeq reagent kit v2 (500 cycles) for the “Neoscreen assay” and a MiSeq reagent kit v2 Micro (300 cycles) for the “CFTR solution”. Bioinformatic analysis was performed with VariFind™ software V.1.0 (Parseq Lab). Individuals who showed one pathogenic allele using the “Neoscreen assay” were re-tested with “CFTR solution”, because the latter included additional intron regions compared to “Neoscreen assay”. Thus 84 individuals were genotyped with “Neoscreen assay”, 81 individuals with “CFTR solution”, and 12 individuals with both of them.

Sanger sequencing was also performed to confirm the identified mutations using the BigDye Terminator v3.1 Cycle Sequencing Kit on a 3500xL Genetic Analyzer (Applied Biosystems, Waltham, MA, USA).

Although both NGS kits were not able to detect major CNVs, except CFTRdele2,3(21kb), we performed multiplex ligation-dependent probe amplification (MLPA) for the heterozygous carriers, individuals with uncertain significance alleles, and those without mutations. For the MLPA, we used SALSA MLPA Probemix P091 CFTR (MRC Holland, Amsterdam, The Netherlands) according to the official protocol. MLPA data analysis was performed using Coffalyzer.net software v.220513.1739 (MRC Holland).

The genetic variants identified in individuals were assessed in terms of their effect on protein structure and/or function using various bioinformatics tools and databases (Annovar, SIFT, Mutation Tester. MutPred; 1000Genomes, Exome Aggregation Consortium, dbSNP, HGMDB, etc.). The pathogenicity of the identified variants was assessed based on recommendations for the interpretation of high-throughput sequencing data [13].

## 3. Results

A total of 35 variants of pathogenic changes in the CFTR gene were detected in the studied sample of patients with CF (Table 2), with both mutations detected in 96.12% of families (124/129). In the total sample of patients from the Republic of Bashkortostan, five mutations were the most frequent (F508del, E92K, 3849+10kbC>T, CFTRdele2.3, L138ins), the total frequency of which was 78.7%. The majority (more than 79%) of patients (103 out of 129) are homozygous or compound-heterozygous for the F508del mutation, with its overall frequency being 54.65%. The highest frequency—85.7%—was found in patients of Ukrainian origin, in 62.7% of Russians, in 46.7% of Tatars, in 40% of Bashkirs, and in one of four Chuvashes patients. In our sample, there were only two chromosomes of Armenian origin and three of Uzbek origin, in which this mutation was also detected, but the small numbers of these groups do not allow us to estimate the true frequency of the mutation in these ethnic groups.

We also assessed the frequency of the complex allele [F508del; L467F] in 74 patients with the F508del variant, which affects the clinical course of cystic fibrosis and the effectiveness of targeted therapy [14,15]. The combination of F508del and L467F variants was found in 16.22% (12 people) of the studied patients, and the frequency of the complex allele was 8.11%.

The frequency of 10 more mutations varied from 0.77 to 1.55%, and the rest were found in a single variant. In addition to the variants known in the literature, we identified a variant, c.3883_3888dup (p.Ile295_Phe1296dup), not described in the literature, as well as a rare variant c.55T>G (W19G). Five variants, including undescribed ones, were not found in other regions of Russia (Table 2).

Statistically significant differences were found in the frequencies of the mutations F508del, E92K, CFTRdele2.3, Y84X, 1525-1G>A, 2184insA, and W1282R, and the complex allele p.[G509D; E217G], compared to those in the registry of patients with cystic fibrosis of the Russian Federation [16].

In five patients, pathogenic changes were detected in a heterozygous state, despite the elevated chloride levels in sweat and the presence of clinical manifestations of the disease. Among these, three patients were of Tatar ethnicity, one of Bashkir ethnicity, and one of Russian origin. To identify causal mutations, it is necessary to study deeper intronic regions, which can only be achieved by whole genome sequencing. Three of these patients had the F508del mutation in a heterozygous state. Their age ranged from 18 to 34 years, and two had a mild pulmonary form of CF, and one had a moderate pulmonary form. One female patient had the E92K variant in a heterozygous state, while the second mutation was not defined. She was 14 years old, with a mixed form of CF and a moderate course, and the diagnosis was established during neonatal screening. The fifth patient (14 years old) was diagnosed with the CFTRdup6b-10 mutation in a heterozygous state; the disease was detected during neonatal screening. Currently, there is a severe course of cystic fibrosis, with a mixed form, diffuse pneumofibrosis, chronic purulent obstructive bronchitis, chronic pancreatic insufficiency, and liver fibrosis associated with cystic fibrosis (F4 according to METAVIR). Complications of the underlying disease are fibrocystic dysplasia of the left lung, chronic respiratory failure I-II (SaO2 < 92%), pulmonary hypertension, osteoporosis, impaired glucose tolerance, and chronic seeding (Cepacia complex ST-709). Based on the clinical characteristics of the patients, further studies are needed to identify a second pathogenic variant of the CFTR gene, especially in minors with severe CF, to enable targeted therapy.

In the three major sub-groups of patients (Russians, Tatars, and Bashkirs), there is a pronounced heterogeneity in the spectrum and frequencies of mutations. In patients of Russian ethnicity, 19 different mutations were identified, in Tatars—17, and in Bashkirs—6. At the same time, only five mutations were common in Russian and Tatar patients; four mutations were also common in Russians and Bashkirs, and only four in Tatars and Bashkirs, despite the fact that the latter belong to the common Turkic group of the Altaic language family.

In patients of Russian ethnicity, nine mutations (F508del, E92K, CFTRdele2-3, 3849+10kbC>T, L138ins, N1303K, S1196X, 2143delT, and Y84X) account for 91.3% of all identified pathogenic changes in the *CFTR* gene. Mutations G551D, 621+1G>T, W19G, S737F, 1717-1G>A, 1367del5, W1282R, 3821delT, and c.2353C>T were identified only in patients of Russian ethnicity (Table 3). In patients of Tatar ethnicity, eight mutations—F508del, E92K, 3849+10kbC>T, 394delTT, S466X (C>G) R1070Q, L138ins, G194R, and G509D—accounted for 87% of all detected variants. Mutations 394delTT, S466X (C>G) R1070Q, G194R, G509D, L1335P, R117C, D1152H, W1310X, 2184insA, and 3041-15T>G were detected only on chromosomes of Tatar origin. In patients of Bashkir ethnicity, three mutations—F508del, E92K, and 3849+10kbC>T—were found on 90% of chromosomes. Mutation 12TG5T was detected only in one patient of Bashkir origin. Along with the F508del mutation, the 2184delA variant was detected in a patient of Ukrainian origin, and the 4015delA variant was found in a patient of Armenian origin. In a patient of Karachay origin, only the W1282X mutation was found in a homozygous state. We also identified a previously undescribed mutation, c.3883_3888dup (p.Ile295_Phe1296dup), on the chromosome of Polish origin.

The obtained results allow us to conclude that there is genetic heterogeneity in the frequency and spectrum of mutations in patients of different ethnic backgrounds living in the Republic of Bashkortostan. The obtained picture fits into the idea that the spectrum of mutations for different populations and geographic groups has a specific pattern, and when making a molecular genetic diagnosis, it is necessary to take it into account in order to simplify and speed up the search for pathogenic variants in a patient, taking into account ethnic and regional background.

## 4. Discussion

### 4.1. General Description

The overall frequency of the F508del (54.65%) is consistent with the literature data. Differences in its frequency in patients of different ethnic origin also conforms with the fact that there are also differences in the prevalence of the F508del mutation in individual regions and populations around the world. In Europe, about 60% of CF patients have at least one F508del mutation. This mutation is more common in southeastern and northern European countries than in their southern counterparts. The frequency of F508del varies from 5% in Georgia to 81.4% in Albania and Denmark [17,18]. This mutation is also the most common in Iran and accounts for 23% of all mutations [19].

Currently, the frequency of the complex allele [F508del;L467F] is being assessed worldwide to predict the effectiveness of targeted therapy. In our study, the frequency of the complex allele was 8.11%, which is higher than the average in Russia, where its frequency is 4.42% [20]. This is probably due to the population genetic characteristics of the studied sample: most of our patients in whom the frequency of the L467F variant was assessed were of Tatar ethnicity (66.67%), and the rest were of Russian origin, which differs from the population structure of the all-Russian cohort of patients.

The second most frequent mutation in the Republic of Bashkortostan, E92K, was found on 13.18% of chromosomes of patients in general, and on three of the four chromosomes of Chuvash origin and 30% of those of Bashkir origin. It was found with a lower frequency in Tatars and Russians, accounting for 16.3% and 7.9%, respectively, and was not detected in patients of other ethnic groups. The *CFTR* variant c.274G>A (p.Glu92Lys) is a change in a conserved nucleotide located in the ABC transporter type 1, transmembrane domain. This variant has been described in the literature, while in vitro functional analysis has shown that it leads to a decrease in chloride transport to less than 10% [21,22] compared to WT. This mutation is a rare variant in most countries of the world, but has been recorded with a frequency of 66% in some populations, for example, in the Chuvash people from Russia [23]. In patients from Turkey, its frequency was 16.7% [24], which is comparable with our data (12.88%), while in Russia, on average, it does not exceed 3.46% [25]. In earlier studies of Turkic-speaking populations of the Volga–Ural region, carriage of the E92K variant was found in Tatars, while it was not detected in Bashkirs [23].

The c.3718-2477C>T (3849+10kbC->T) variant involves a non-conserved deep intronic nucleotide change. This mutation creates a new splice donor site within intron 22 of the *CFTR* gene, resulting in the insertion of a new disrupted exon of 84 nucleotides, the formation of an in-frame stop codon, and the subsequent formation of a truncated non-functional CFTR protein [24]. This variant has been reported in several CF patients with a mild phenotype, including at least three homozygotes [26,27], and has been included in the ACMG CFTR list [28]. This variant is present in databases (rs75039782, gnomAD 0.3%) and is included in the CF variant panel of the American College of Medical Genetics [22,28,29]. c.3718-2477C>T is the seventh most common CFTR mutation in the United States and the eighth in Europe, with over 1400 CF patients worldwide carrying it [30]. This mutation is widespread in some populations, such as Ashkenazi Jews and CF patients in Latvia and Poland [31]. In Russia, it is the fifth most common (2.22%) (Table 2), while in the studied region it is the third most common variant (4.64%), with the highest frequency detected in patients of Bashkir ethnicity (10%). Variant 3849+10kbC->T is the most common “mild” pathogenic variant in the world, identified on 12 chromosomes in our samples, and is represented as a compound heterozygous variant. Clinically, patients have a questionable sweat test and negative neonatal screening, so the diagnosis was made later, more often at the age of 6–7 years. Male patients do not suffer from azoospermia, as with other pathogenic variants in the *CFTR* gene. For example, one 35-year-old patient from the Republic of Bashkortostan has two children. In one patient, the mild course of the disease is characterized by borderline indicators of sweat chlorides.

Pathogenic variant CFTRdele2,3(21 kb) is a 21,080 bp deletion that results in the loss of exons 2 and 3 in *CFTR* mRNA, resulting in a premature termination signal in exon 4. Among Russian CF patients, this mutation is the second most common, but in our study its overall frequency was 3.48% and it was detected only in patients of Russian ethnicity. In this population, its frequency reached 6.5% and it was the third most common mutation in Russian patients from the Republic of Bashkortostan and the fourth most common in the total sample. This pathogenic variant is characterized by an “East Slavic” origin [32], which is consistent with our results. The relative frequency of the CFTRdele2-3 mutation is highest in CF patients from Russia, decreasing in Europe from east to west and south: Czech Republic—4.7%, and Poland and Ukraine—1%. This was discussed at a joint meeting of the World Health Organization (WHO) in 2002. The CFTRdele2-3 mutation was detected in seven patients in a compound heterozygous state, five of them with the F508del/CFTRdele2-3 genotype, characterized by moderate to severe course, early manifestation, and high chloride levels in sweat up to 200 mmol/L; these patients were identified during neonatal screening. There is marked, severe damage to the respiratory and gastrointestinal tract with an unfavorable prognosis. There is also a case with the CFTRdele2-3/3883_3888dup genotype (CFTRdele2-3 was inherited from a Russian parent).

The fifth most frequent pathogenic mutation in the studied sample was c.413_415dupTAC (also known as p.L138dup and L138ins, rs397508679). It is a mild pathogenic variant located in the coding exon 4 of the *CFTR* gene, which results in duplication of an additional amino acid residue between codons 138 and 139. This variant was found on 1.8% of chromosomes of Russian patients [33], which is lower than in our patient sample, where it was found in patients of Russian and Tatar ethnicity, reaching 2.71% overall. Previously, this mutation was identified in a Polish man with congenital bilateral absence of the vas deferens (CBAVD) in combination with the 5T allele [34]. This alteration was also described in an individual with CF in combination with the c.3717+12191C>T mutation [35]. Based on structural analysis, this pathogenic variant is suggested to result in a significant decrease in structural stability [36]. Furthermore, CF human bronchial epithelial (CFBE) cells stably expressing this variant only exhibited 1.6% function compared to wild-type CFTR compared to cells expressing the wild-type CFTR protein [37]. The worldwide frequency of this duplication is approximately 0.0004% (1/250744) in the gnomAD database [38].

It is known that the worldwide frequency of common CFTR gene mutations, except for F508del, is relatively low, and most of them are geographically limited to small areas [39]. This is also correct for their occurrence in the Republic of Bashkortostan, as some of these mutations were found only in certain populations and were not identified in others.

### 4.2. Major Sub-Groups Specificity

Twelve mutations associated with CF were identified in individuals of Tatar ethnicity, which were not found in our sample in representatives of other ethnic groups: 394delTT, S466X (C>G), R1070Q, G194R, L1335P (c. 4004T>C), R117C, D1152H, 1525-1G>A, 2184insA, 3041-15T>G, W1310X, and CFTRdup6b-10. Of these, mutations 394delTT, S466X (C>G), R1070Q, and G194R are found on more than one chromosome. Moreover, mutation R1070Q in our sample was present only in the cis position with mutation S466X. Previously, cases were described where the R1070Q mutation was present on the chromosome separately from S466X. According to the literature, all patients with the complex R1070Q-S466X allele had pancreatic insufficiency, while, if only the R1070Q mutation was present, it led to a milder form of the disease (e.g., CBAVD) [40].

Variant 394delTT (c.262_263delTT) was previously considered the second most common in the Republic of Bashkortostan when only a targeted list of variants was analyzed that had been tailored for the general Russian population, but with only the variants common in Russia [41]. According to our study, its frequency in the families from the studied region is lower (1.55%). This is one of the variants common in Northern Europe, which was also previously identified in Russian patients with CF (but not with CVABD syndrome) [33]. This mutation is located in the coding exon 3 of the *CFTR* gene.It is the result of a two-nucleotide deletion at positions 262–263, which causes a frameshift with the appearance of an alternative stop codon (p.L88Ifs*22). This mutation is associated with increased sweat chloride levels, pancreatic insufficiency, and decreased lung function [22]. The frequency of this mutation was reported to be 8.5% in a cohort of 331 CF patients from Sweden [42]. It was found in a homozygous state in an infant with severe manifestations of cystic fibrosis, including pancreatic insufficiency, anemia, developmental delay, elevated sweat chloride levels, and severe lung disease [43]. In addition to the clinical data presented in the literature, this alteration is proposed to result in loss of function due to premature truncation of the protein or nonsense-mediated mRNA decay.

As for the G194R mutation, although it was detected only on two chromosomes, it has a very low frequency in the general Russian population (Table 2). Meanwhile, this variant is well known and its biochemical characteristics have been previously obtained. It reduces, but does not completely stop, the production of mature CFTR and can be rescued by the tested modulators [44]. In a single case, the W1310X variant was detected in Tatars, which was previously described in seven unrelated patients from Russia [2]. There are 28 patients with this variant in the CFTR2 database.

In the Russian population, in addition to the generally common CFTRdele2.3 mutation, 11 more mutations (12 in total) were identified that were not found in anyone else in the Republic of Bashkortostan sample: N1303K, S1196X, G551D, 621+1G>T, W19G, S737F, 1717-1G>A, 1367del5, W1282R, 3821delT, and c.2353C>T.

Like CFTRdele2.3, the missense variant c.3909C>G (p.Asn1303Lys, N1303K) is a well-known, widely described pathogenic variant that accounts for approximately 1.3% of all CF-associated mutations, and is often associated with the classic cystic fibrosis phenotype [45]. According to the Cystic Fibrosis Mutation Database [46], the p.Asn1303Lys variant is the fourth most common CFTR variant worldwide, and the Clinical and Functional Translation of CFTR (CFTR2) database [46] reports the p.Asn1303Lys variant in more than 3300 patients. The p.Asn1303Lys variant was also reported in five patients with congenital bilateral absence of the vas deferens (CBAVD) [47,48], and in four patients with chronic pancreatitis [47,49]. The frequency of the N1303K mutation in our study was 1.55%, which is comparable with the all-Russian prevalence (Table 2).

The S1196X mutation is a premature stop codon and is one of the most common mutations of this type in the studied sample of Russians, although generally its allelic frequency is quite low (0.01%). This is lower than the frequency of mutation W1282X, which is also belongs to the same type of mutation [50].

However, unlike S1196X, the W1282X mutation is typical for CF patients from the North Caucasus populations. In particular, in our sample, one of the patients with this mutation in a homozygous state was of Karachay ethnic origin. Most likely, the patient from the Russian sample has ancestors from populations representing the Caucasus region, which once again indicates the heterogeneity of this sample.

It is the heterogeneity of the Russian and Tatar populations that can explain the increased diversity of mutations compared to other populations of the Republic of Bashkortostan. Only in representatives of these two ethnic groups was a rare mutation, Y84X, detected. It has not been previously described worldwide, although it is present in the Russian registry; the ethnicity of the patient from the registry however is unknown.

In the Russian population from the studied region, the deletions c.3691del, also known as CF3821delT, and 1367del5 are also of interest. 3821delT causes a premature translational termination (p.Ser1231Profs*4), which was observed in people with cystic fibrosis [51,52,53]. This variant is absent in population databases (gnomAD has no frequency) and it is present in the Russian population with a frequency comparable to the frequency in our sample (Table 2). Mutation 1367del5 (c.1243_1247delAACAA, p.Asn415X) leads to the formation of a premature termination codon. The variant was absent on the chromosomes of individuals from the control sample (*n* = 247,414) (gnomAD). c.1243_1247delAACAA and a variant causing similar premature termination have been reported in the literature in individuals with cystic fibrosis [2,54]. This variant was identified in one patient with clinically confirmed cystic fibrosis and was classified in the context of the variant reclassification project in the German Cystic Fibrosis Registry [55].

Only six mutations were identified in the Bashkir population, three of which are common in the region (F508del, E92K, and 3849+10kbC>T). The G509D mutation was also found in Tatars, and 2143delT was found in Russians. Additionally, a mutation was identified that was not detected in any other population in the region except the Bashkirs (12TG5T).

G509D was previously described as a complex allele with the E217Gp.[E217G;G509D] substitution in a patient from Russia with the F508del/[E217G;G509D] genotype. The pathogenicity of this complex variant and the possibility of therapy were also assessed [56]. In our sample, two patients had the F508del/[E217G;G509D] genotype, and two patients had the previously undescribed E92K/[E217G;G509D] genotype. At the same time, in the population sample from the Republic of Bashkortostan, we also identified nine samples with the E217G variant, one of which was a carrier of the F508del mutation, and eight more had no known pathogenic mutations.

It was shown that the frequency of 2143delT exceeds 2% in the general Russian population (although it is currently presented at a lower frequency in patients according to the Registry), and thus can be referred to as a frequent one in this ethnic group [2]. In Latvia, this variant combined with the W1282R mutation displayed a classic CF phenotype with pancreatic insufficiency [57]. In our sample, one patient with Bashkir origin also had Russian ancestry and had the F508del/2143delT genotype, while a Russian patient had this mutation in a homozygous state. The child with the homozygous 2143delT genotype has been ill since birth. The diagnosis was made in 2004 at the age of 6 months based on respiratory and intestinal syndrome, and high sweat chlorides of 185 mmol/L, 115 mmol/L, and 174 mmol/L; cystic fibrosis, mixed form, severe course; chronic bronchitis, moderate exacerbation; bronchiectasis on both sides; and chronic pancreatic insufficiency. Complications were liver fibrosis; secondary pulmonary hypertension stage I; and secondary osteoporosis. Physical development was delayed and BMI was 16.9 kg/m^2^.

As for the patient with the F508del/2143delT genotype, he was diagnosed with cystic fibrosis of the pulmonary form, of moderate severity at the age of 11, with the following: concomitant: sphincter of Oddi dysfunction of the pancreatic type. Microbiological diagnosis: chronic seeding of Ps. aeruginosa. Sweat sample on the Nanoduct: 133–116 mmol/L.

### 4.3. Rare Variants

The 12TG5T mutation is absent in the latest version of the Cystic Fibrosis Registry in the Russian Federation for 2021. This mutation in intron 9 of the CFTR gene is a variant of the TG[n]T[m] polymorphic region adjacent to exon 10. Pathogenicity for this variant remains unclear. Studies have shown that longer TG repeat sizes (TG11, 12, and 13) in individuals with T[5] have a higher susceptibility to the disease than those with smaller TG repeat sizes when present in trans with a pathogenic CFTR variant [58,59,60]. Associated symptoms associated with CF include congenital bilateral absence of the vas deferens (CBAVD), male infertility, and mild or classic forms of cystic fibrosis, the severity of which depends on the CF variant on the opposite allele [61]. In conclusion, the 12TG5T variant is most likely pathogenic, although further studies are needed to fully establish its clinical significance. According to the CFTR2 database, patients with this variant in a compound heterozygous state both had and did not have a diagnosis of CF. In our study, the 12TG5T variant (c.1210-11T>G) was detected in a 20-year-old patient of Bashkir origin in combination with the 394delTT mutation with a severe course of the disease. The boy has been ill since birth: cough since the 1st month, bloating, poor weight gain, and fatty profuse stool with an abundance of gases. The patient has chronic respiratory failure, moderate chronic pancreatic insufficiency, compensated liver cirrhosis, Child–Pugh class A, portal hypertension syndrome, intrahepatic form, splenomegaly, and communicating hydrocele of the right testicle.

We also detected a single case of the c.1585-1G>A variant, also known as c.1717-1G>A, which was found in combination with F508del in a Russian patient. This is a mutation in a canonical splice acceptor site and therefore affects transcription of the normal gene product. The c.1585-1G>A variant is listed by ACMG as part of the panel recommended for routine diagnosis and carrier testing of cystic fibrosis [28]. The c.1585-1G>A variant is one of the ten most common CFTR variants in individuals of Northern European descent, accounting for 0.6% of the disease, and is associated with the classic cystic fibrosis phenotype [62]. It was found in 635/79392 disease-associated alleles in people from North America and Europe [22]. Our 9-year-old patient has classic clinical manifestations of CF, mixed form, moderate course, chronic respiratory failure, and chronic pancreatic insufficiency. The disease was detected during neonatal screening (IRT test, 138 ng/mL; retest, 311.9 ng/mL; and increased sweat chloride content, 103.112 mmol/L).

Variants S737F, 3041-15T>G, W19G, and c.3883_3888dup are not registered in the latest version of the Cystic Fibrosis Patient Registry in the Russian Federation for 2021 and have not been described in other patients from Russia.

Variant S737F was detected in a 15-year-old patient of Russian ethnicity in combination with the F508del mutation. The disease was detected during neonatal screening (IRT test, 145 ng/mL; retest, 270 ng/mL; sweat chlorides, 64.69 mmol/L), with mixed cystic fibrosis and moderate course. The p.S737F variant (also known as c.2210C>T) is located in coding exon 14 of the CFTR gene and replaces serine, which is neutral and polar, with phenylalanine, which is neutral and non-polar, at codon 737 of the CFTR protein (p.Ser737Phe). This variant has been identified in several individuals with a second CFTR variant and intermediate sweat chloride levels [63,64,65]. It was also earlier identified in the homozygous state in one child with intermediate sweat chloride levels and in association with a gross deletion in a child with elevated sweat chloride levels; both infants developed hypochloremicalkalosis [65]. Based on currently available data, the clinical significance of this alteration remains unclear. ClinVar contains an entry for this variant (variant code: 53454). This variant was identified in three unrelated patients with clinically confirmed cystic fibrosis as part of a project to reclassify variants in the German Cystic Fibrosis Registry [55].

Variant 3041-15T>G was identified in a female patient of Tatar ethnicity born in 2012 in combination with the L138ins mutation. The 12-year-old patient has a mild, uneventful course of the disease, accompanied by arthralgia and urinary tract infections. The sequence change 3041-15T>G (c.2909-15T>G) occurs in intron 17 of the *CFTR* gene. This variant is present in population databases (rs397508455, ExAC 0.006%). ClinVaralso contains a record for this variant (variation ID: 53592). Experimental data demonstrated that the variant affects mRNA splicing; in particular, the variant was found to result in aberrant splicing in 81.23% of the assessed transcripts, resulting in out-of-frame skipping of exon 16 [66]. It was found at a frequency of 4 × 10^−6^ in 250,448 control chromosomes (gnomAD), and has been described in the literature in newborn screening cases associated with cystic fibrosis (CF) and CFTR-related metabolic syndrome [7,67,68,69]; it was also described in two infants with an initial inconclusive diagnosis of CF, one of whom was diagnosed with CF by school age and the other remained inconclusive during the same follow-up period [70,71]. c.2909-15T>G was found to be homozygous in one male affected by congenital bilateral absence of the vas deferens (CBAVD) [72] and has been described in the literature as associated with CBAVD and frequently occurring in infertile males [73,74]. Eight applicants have submitted clinical significance assessments for this variant to ClinVar since 2014 and classified the variant as pathogenic (*n* = 2)/likely pathogenic (*n* = 5) or VUS (*n* = 2). Based on the evidence outlined above, the variant was classified as likely pathogenic. Considering that in our patient the 3041-15T>G change is combined with the L138ins variant (c.413_415dupTAC, rs397508679, p.Leu138dup), which is a mild pathogenic variant and is classified as a class IV-V mutation in the *CFTR* gene by the pathogenicity mechanism, the girl does not have pronounced manifestations of CF and is under dynamic observation.

Variant W19G (c.55T>G) in our sample is found on one chromosome in a heterozygous state with the F508del mutation. Previously, this variant was also described in a compound-heterozygous state with F508del in a patient with cystic fibrosis with azoospermia [75]. In our study, a 32-year-old male patient has been ill since birth, with a mixed form of cystic fibrosis, moderate course, and increased sweat chloride content of 111–123 mmol/L. However, he moved to another region and there are no data on his current health status.

For the first time, in a patient of Polish origin, we identified the mutation c.3883_3888dup, leading to duplication p.Ile1295_Phe1296dup in a compound-heterozygous state with the CFTRdele2-3 mutation. In this region, the c.3883_3886del deletion was previously described, which leads to a frameshift with a predicted alternative stop codon (p.I1295Ffs*32) and was detected in patients with cystic fibrosis [35,76], but the pathogenicity of the identified duplication is a subject for further study. In the 19-year-old patient from our sample, this change is combined with the CFTRdele2-3 mutation. There are no neonatal screening data. The diagnosis of cystic fibrosis was established at 7 years old based on respiratory and intestinal syndrome, and high sweat chlorides of 102–118 mmol/L. Currently, a mixed form of the disease is observed, with a moderate course, bronchiectasis S5, S8 on the left, chronic respiratory failure, chronic pancreatic insufficiency, and chronic seeding of *Ps. aeruginosa*.

## 5. Conclusions

In total, 35 variants of the *CFTR* gene with pathogenic/probably pathogenic significance were identified. In 96.12% of families, both mutations were detected. Thirteen variants occurred with a frequency of more than 1%, in total making up 88.76% of all identified changes. Genetic heterogeneity in the frequency and spectrum of mutations was found in patients of different ethnicity living in the Republic of Bashkortostan. Mutations G551D, 621+1G>T, W19G, S737F, 1717-1G>A, 1367del5, W1282R, 3821delT, and c.2353C>T were detected only in patients of Russian origin; mutations 394delTT, S466X (C>G) R1070Q, G194R, G509D, L1335P, R117C, D1152H, W1310X, 2184insA, and 3041-15T>G were detected only on chromosomes of Tatar origin; and mutation 12TG5T was detected only in a patient of Bashkir origin. A patient of Karachay origin was found to have the W1282X mutation in a homozygous state, and a previously undescribed mutation c.3883_3888dup (p.Ile295_Phe1296dup) was found on a chromosome of Polish origin.

Thus, the local genetic diversity is a background of the mutation distribution pattern. The main set of the *CFTR* mutations in the Republic of Bashkortostan of Russia is established though some rare mutations that occur in separate patients, and it is crucial to perform a thorough search for mutations in patients of different geographic origin to determine the molecular genetic basis of each individual case of the disease.

Taking into account the genetic pattern of certain populations highlighting the genetic specificity of the region promotes a more efficient search for common *CFTR* mutations in patients. This approach is convenient when performing a search for certain mutations, e.g., by selecting a suitable diagnostic kit for both screening and establishing a correct molecular genetic diagnosis before the use of NGS sequencing, especially when it is combined with neonatal screening, which is currently conducted in the Republic of Bashkortostan as an advanced program.

## Figures and Tables

**Table 1:** Ethnic composition of families of patients with cystic fibrosis in the Republic of Bashkortostan.

#	Ethnic Groups	Language Affiliation	*n*	Frequency, %
1	Russians	Slavic	56	43.4
2	Tatars	Turkic	38	29.5
3	Bashkirs	Turkic	7	5.4
4	Ukrainians	Slavic	4	3.1
5	Chuvashes	Turkic	1	0.8
6	Armenians	Armenian	1	0.8
7	Karachays	Turkic	1	0.8
8	Admixed		21	16.3
Total				100

**Table 2:** Spectrum and frequency of pathogenic changes in the nucleotide sequence of the CFTR gene in patients with cystic fibrosis from the Republic of Bashkortostan. The frequencies are calculated by individual families.

	Legacy Name	cDNA	Protein	RB		RF **		*p*
				*n*	%	*n*	%	
1	F508del	c.1521_1523del	p.Phe508del	141	54.65	3829	51.55	<0.001
2	E92K	c.274G>A	p.Glu92Lys	34	13.18	257	3.46	<0.001
3	3849+10kbC>T	c.3718-2477C>T	-	12	4.64	165	2.22	0.495
4	CFTRdele2.3	c.54-5940_270+10250del21kb	p.Ser18ArgfsX16	9	3.48	454	6.11	<0.001
5	L138ins	c.413_415dupTAC	p.Leu138dup	7	2.71	122	1.64	0.745
6	N1303K	c.3909C>G	p.Asn1303Lys	4	1.55	113	1.52	0.301 *
7	394delTT	c.262_263del	p.Leu88IlefsTer22	4	1.55	63	0.85	0.833 *
8	p.[G509D; E217G]	[c.1526G>A; c.650A>G]	p. [Gly509Asp; p.Glu217Gly]	3	1.16	1	0.01	<0.001
9	2143delT	c.2012del	p.Leu671Ter	3	1.16	147	1.98	0.050 *
10	p. [S466X; R1070Q]	[c.1397C>G; c.3209G>A]	[p.Ser466Ter; p.Arg1070Gln]	3	1.16	34	0.46	0.609 *
11	S1196X	c.3587C>G	p.Ser1196Ter	3	1.16	32	0.43	0.797 *
12	W1282X	c.3846G>A	p.Trp1282Ter	3	1.16	128	1.72	0.096 *
13	Y84X	c.252T>A	p.Tyr84Ter	3	1.16	4	0.05	0.002 *
14	G194R	c.580G>A	p.Gly194Arg	2	0.77	5	0.07	0.091 *
15	1525-1G>A	c.1393-1G>A	-	2	0.77	1	0.01	0.002 *
16	G551D	c.1652G>A	p.Gly551Asp	1	0.39	3	0.04	0.593 *
17	2184delA	c.2052delA	p.Lys684Asnfs*38	1	0.39	2	0.03	0.445 *
18	621+1G>T	c.489+1G>T	-	1	0.39	14	0.19	0.654 *
19	L1335P (4136T>C)	c.4004T>C	p.Leu1335Pro	1	0.39	13	0.18	0.692 *
20	R117C (481C>T)	c.349C>T	p.Arg117Cys	1	0.39	7	0.09	0.987 *
21	4061G>A (W1310X)	c.3929G>A	p.Trp1310Ter	1	0.39	23	0.31	0.977 *
22	D1152H (3586G>C)	c.3454G>C	p.Asp1152His	1	0.39	9	0.12	0.884 *
23	1717-1G>A	c.1585-1G>A	-	1	0.39	4	0.05	0.716 *
24	1367del5	c.1243_1247delAACAA	p.Asn415Ter	1	0.39	31	0.42	0.737 *
25	3821delT (S1231fs)	c.3691delT	p.Ser1231ProfsX4	1	0.39	38	0.51	0.555 *
26	4015delA	c.3883delA	p.Ile1295PhefsX33	1	0.39	13	0.18	0.692 *
27	2184insA	c.2052_2053insA	p.Gln685ThrfsX4	1	0.39	144	1.94	0.010 *
28	W1282R	c.3844T>C	p.Trp1282Arg	1	0.39	128	1.72	0.018 *
29	CFTRdup6b-10	c.(743+1_744-1)_(1584+1_1585-1)dup	-	1	0.39	10	0.13	0.829 *
30	2485C>T (R785X)	c.2353C>T	p.Arg785X	1	0.39	12	0.16	0.734 *
31	S737F	c.2210C>T	p.Ser737Phe	1	0.39	-	-	0.067 *
32	-	c.3883_3888dup	p.Ile1295_Phe1296dup	1	0.39	-	-	0.067 *
33	3041-15T>G	c.2909-15T>G	-	1	0.39	-	-	0.067 *
34	12TG5T	c.1210-11T>G	-	1	0.39	-	-	0.067 *
35	W19G	c.55T>G	p.Trp19Gly	1	0.39	-	-	0.067 *
36	Not identified (X)			5	1.94	377	9.5%	<0.001
Number of chromosomes				258	100			

* Fisher Exact χ^2^ Test with Yates’ Correction; ** According to the Registry of Patients with Cystic Fibrosis in the Russian Federation (2021), the mutation rate was calculated based 3965 patients.

**Table 3:** Spectrum and occurrence of causal variants in the *CFTR* gene by ethnic ancestry.

z	Mutation	Russians 126		Tatars 92		Bashkirs 20		Ukrainians 8		Chuvashes 4		Armenians 2		Uzbeks 2		Belorussian 1		Karachays 2		Poles 1	
1	F508del	79	0.627	43	0.467	8	0.400	7	0.875	1	0.250	1	0.500	1	0.500	1	1.000	0	0.000	0	0.000
2	E92K	10	0.079	15	0.163	6	0.300	0	0.000	3	0.750	0	0.000	0	0.000	0	0.000	0	0.000	0	0.000
3	CFTRdele2-3	9	0.071	0	0.000	0	0.000	0	0.000	0	0.000	0	0.000	0	0.000	0	0.000	0	0.000	0	0.000
4	3849+10kbC>T	1	0.008	9	0.098	2	0.100	0	0.000	0	0.000	0	0.000	0	0.000	0	0.000	0	0.000	0	0.000
5	L138ins	5	0.040	2	0.022	0	0.000	0	0.000	0	0.000	0	0.000	0	0.000	0	0.000	0	0.000	0	0.000
6	N1303K	4	0.032	0	0.000	0	0.000	0	0.000	0	0.000	0	0.000	0	0.000	0	0.000	0	0.000	0	0.000
7	S1196X	3	0.024	0	0.000	0	0.000	0	0.000	0	0.000	0	0.000	0	0.000	0	0.000	0	0.000	0	0.000
8	394delTT	0	0.000	4	0.043	0	0.000	0	0.000	0	0.000	0	0.000	0	0.000	0	0.000	0	0.000	0	0.000
9	S466X(C>G), R1070Q	0	0.000	3	0.033	0	0.000	0	0.000	0	0.000	0	0.000	0	0.000	0	0.000	0	0.000	0	0.000
10	G194R	0	0.000	2	0.022	0	0.000	0	0.000	0	0.000	0	0.000	0	0.000	0	0.000	0	0.000	0	0.000
11	G551D	1	0.008	0	0.000	0	0.000	0	0.000	0	0.000	0	0.000	0	0.000	0	0.000	0	0.000	0	0.000
12	G509D	0	0.000	2	0.022	1	0.050	0	0.000	0	0.000	0	0.000	0	0.000	0	0.000	0	0.000	0	0.000
13	2184delA	0	0.000	0	0.000	0	0.000	1	0.125	0	0.000	0	0.000	0	0.000	0	0.000	0	0.000	0	0.000
14	621+1G>T	1	0.008	0	0.000	0	0.000	0	0.000	0	0.000	0	0.000	0	0.000	0	0.000	0	0.000	0	0.000
15	L1335P(c.4004T>C)	0	0.000	1	0.011	0	0.000	0	0.000	0	0.000	0	0.000	0	0.000	0	0.000	0	0.000	0	0.000
16	R117C	0	0.000	1	0.011	0	0.000	0	0.000	0	0.000	0	0.000	0	0.000	0	0.000	0	0.000	0	0.000
17	W19G	1	0.008	0	0.000	0	0.000	0	0.000	0	0.000	0	0.000	0	0.000	0	0.000	0	0.000	0	0.000
18	S737F	1	0.008	0	0.000	0	0.000	0	0.000	0	0.000	0	0.000	0	0.000	0	0.000	0	0.000	0	0.000
19	2143delT	2	0.016	0	0.000	1	0.050	0	0.000	0	0.000	0	0.000	0	0.000	0	0.000	0	0.000	0	0.000
20	D1152H	0	0.000	1	0.011	0	0.000	0	0.000	0	0.000	0	0.000	0	0.000	0	0.000	0	0.000	0	0.000
21	1717-1G>A	1	0.008	0	0.000	0	0.000	0	0.000	0	0.000	0	0.000	0	0.000	0	0.000	0	0.000	0	0.000
22	1525-1G>A	0	0.000	1	0.011	0	0.000	0	0.000	0	0.000	0	0.000	1	0.500	0	0.000	0	0.000	0	0.000
23	1367del5	1	0.008	0	0.000	0	0.000	0	0.000	0	0.000	0	0.000	0	0.000	0	0.000	0	0.000	0	0.000
24	4015delA	0	0.000	0	0.000	0	0.000	0	0.000	0	0.000	1	0.500	0	0.000	0	0.000	0	0.000	0	0.000
25	2184insA	0	0.000	1	0.011	0	0.000	0	0.000	0	0.000	0	0.000	0	0.000	0	0.000	0	0.000	0	0.000
26	Y84X	2	0.016	1	0.011	0	0.000	0	0.000	0	0.000	0	0.000	0	0.000	0	0.000	0	0.000	0	0.000
27	c.3883_3888dup	0	0.000	0	0.000	0	0.000	0	0.000	0	0.000	0	0.000	0	0.000	0	0.000	0	0.000	1	1.000
28	3041-15T>G	0	0.000	1	0.011	0	0.000	0	0.000	0	0.000	0	0.000	0	0.000	0	0.000	0	0.000	0	0.000
29	W1282X	1	0.008	0	0.000	0	0.000	0	0.000	0	0.000	0	0.000	0	0.000	0	0.000	2	1.000	0	0.000
30	12TG5T	0	0.000	0	0.000	1	0.050	0	0.000	0	0.000	0	0.000	0	0.000	0	0.000	0	0.000	0	0.000
31	W1310X	0	0.000	1	0.011	0	0.000	0	0.000	0	0.000	0	0.000	0	0.000	0	0.000	0	0.000	0	0.000
32	W1282R	1	0.008	0	0.000	0	0.000	0	0.000	0	0.000	0	0.000	0	0.000	0	0.000	0	0.000	0	0.000
33	3821delT	1	0.008	0	0.000	0	0.000	0	0.000	0	0.000	0	0.000	0	0.000	0	0.000	0	0.000	0	0.000
34	CFTRdup6b-10	0	0.000	1	0.011	0	0.000	0	0.000	0	0.000	0	0.000	0	0.000	0	0.000	0	0.000	0	0.000
35	c.2353C>T	1	0.008	0	0.000	0	0.000	0	0.000	0	0.000	0	0.000	0	0.000	0	0.000	0	0.000	0	0.000
36	x	1	0.008	3	0.033	1	0.050	0	0.000	0	0.000	0	0.000	0	0.000	0	0.000	0	0.000	0	0.000
37	*n*	126	1.000	92	1.000	20	1.000	8	1.000	4	1.000	2	1.000	2	1.000	1	1.000	2	1.000	1	1.000

## Data Availability

The data presented in this study are available on request from the corresponding author due to due to privacy or ethical restrictions.
